# Mechanism of *Lactiplantibacillus plantarum* regulating Ca^2+^ affecting the replication of PEDV in small intestinal epithelial cells

**DOI:** 10.3389/fmicb.2023.1251275

**Published:** 2023-09-26

**Authors:** Zifei Kan, Shujuan Zhang, Guisong Liao, Zheng Niu, Xiangyang Liu, Zhiwei Sun, Xia Hu, Yiling Zhang, Shasha Xu, Jingyi Zhang, Hong Zou, Xingcui Zhang, Zhenhui Song

**Affiliations:** ^1^College of Veterinary Medicine, Southwest University, Chongqing, China; ^2^School of Medicine, University of Electronic Science and Technology of China, Chengdu, China; ^3^College of Veterinary Medicine, Northwest A and F University, Shanxi, China; ^4^College of Veterinary Medicine, Xinjiang Agricultural University, Ürümqi, China; ^5^College of Animal Scienceand Technology, Chongqing Three Gorges Vocational College, Chongqing, China; ^6^Immunology Research Center, Medical Research Institute, Southwest University, Chongqing, China

**Keywords:** *Lactiplantibacillus plantarum*, LP-1S, PEDV, Ca^2+^, intestinal epithelial cells

## Abstract

Porcine epidemic diarrhea virus (PEDV) mainly invades the small intestine and promotes an inflammatory response, eventually leading to severe diarrhea, vomiting, dehydration, and even death of piglets, which seriously threatens the economic development of pig farming. In recent years, researchers have found that probiotics can improve the intestinal microenvironment and reduce diarrhea. At the same time, certain probiotics have been shown to have antiviral effects; however, their mechanisms are different. Herein, we aimed to investigate the inhibitory effect of *Lactiplantibacillus plantarum* supernatant (LP-1S) on PEDV and its mechanism. We used IPEC-J2 cells as a model to assess the inhibitory effect of LP-1S on PEDV and to further investigate the relationship between LP-1S, Ca^2+^, and PEDV. The results showed that a divalent cation chelating agent (EGTA) and calcium channel inhibitors (Bepridil hydrochloride and BAPTA-acetoxymethylate) could inhibit PEDV proliferation while effectively reducing the intracellular Ca^2+^ concentration. Furthermore, LP-1S could reduce PEDV-induced loss of calcium channel proteins (TRPV6 and PMCA1b), alleviate intracellular Ca^2+^ accumulation caused by PEDV infection, and promote the balance of intra- and extracellular Ca^2+^ concentrations, thereby inhibiting PEDV proliferation. In summary, we found that LP-1S has potential therapeutic value against PEDV, which is realized by modulating Ca^2+^. This provides a potential new drug to treat PEDV infection.

## 1. Introduction

Porcine epidemic diarrhea (PED) is an acute, highly contagious intestinal disease of pigs caused by Porcine epidemic diarrhea virus (PEDV) (Cao et al., 2015). The virus mainly colonizes the intestinal mucosa of the host, with the largest amount of virus found in the small intestine mucosa, but can also be detected in the lung, heart, and liver (Narayanan et al., 2012). The clinical signs of the disease are vomiting and watery diarrhea, which consequently lead dehydration in newborn piglets, eventually leading to their death (Ding et al., 2014). PED is more frequent in winter when it is cold, and has a very high mortality rate for piglets within 2 weeks of age, causing serious economic losses to the pig industry (Wu et al., 2020). PED outbreaks caused by highly virulent PEDV variants are still occurring in pig farms in several countries or regions, and existing vaccines or drugs cannot achieve the desired control of PED. Moreover, the continuous detoxification of sick pigs poses additional challenges to the prevention and control of this disease (Jang et al., 2022; Yao et al., 2023). Therefore, there is an urgent need to develop new drugs for this infectious disease.

Ca^2+^ acts as an important second messenger in intracellular signal transduction processes. Ca^2+^ is essential for the transmembrane transmission of biological signals, the regulation of glandular secretion, and the maintenance of acid-base homeostasis in the body (Seo et al., 2015; Hussey et al., 2023). The maintenance of calcium homeostasis depends on various calcium channel proteins [e.g., transient receptor potential cation channel subfamily V member 6 (TRPV6) and stromal interaction molecule (STIM)] in the membranes of the endoplasmic reticulum, Golgi apparatus, mitochondria, and other cellular organelles (Liou et al., 2005; Bibollet et al., 2023), transporters [sodium-calcium exchanger Na(+)/Ca(2+)-Exchange Protein 1 (NCX-1) (Tarifa et al., 2022)], and ion pumps [e.g., the cell membrane calcium pump, plasma membrane calcium-transporting ATPase 1 (PMCA1b)] (Rodrat et al., 2022; Dai et al., 2023). Disruption of any of these components can affect calcium homeostasis. Under normal conditions, the concentration of free intracellular Ca^2+^ is much lower than the concentration of Ca^2+^ in other calcium pools, such as the endoplasmic reticulum and outside the cell (Peng et al., 2001). In general, the transcellular pathway for intestinal Ca^2+^ uptake consists of three steps (Guo et al., 2016): (i) Crossing the brush border membrane (BBM) of the intestinal cells via the epithelial Ca^2+^ channel (TRPV6); (ii) binding to proteins with high Ca^2+^ affinity to form calcium-binding protein-D9k (CaBPD9k), which in turn moves from the BBM to the basolateral membrane (BLM); and (iii) extracellular excretion via the plasma membrane Ca^2+^-ATPase (PMCA1b/Ca^2+^ pump) and Na^+^/Ca^2+^ exchanger (NCX1). Among them, TRPV6 and PMCA1b, as calcium channel proteins, have received much research attention because they are the major Ca^2+^ transport proteins in the intestinal epithelium and have important roles in maintaining intra- and extracellular homeostasis and controlling ions entering and leaving the cell (Zhao et al., 2022).

In recent years, research has shown that a variety of probiotics can maintain microbial homeostasis in the intestinal environment and prevent diarrhea (Hu et al., 2022; Liang et al., 2023). Antiviral research into probiotics and their metabolites has also become a hot topic (Leblanc et al., 2022; Liu et al., 2022) *Lactiplantibacillus plantarum* (Lp) significantly reduced the cytopathic effects of respiratory syncytial virus (RSV), influenza B virus, and HCoV-229E coronavirus (Spacova et al., 2023). Lp is a gram-positive facultative anaerobic bacterium that is widely found in nature, is commonly found in various fermented foods, and is one of the important flora of the gut microbiota. Its optimum growth temperature is 30 ~ 37°C, its optimum pH is about 7.0, and the colony growth shape is white opaque dots on MRS (De Man, Rogosa and Sharpe) solid medium. A strain of *Lactobacillus plantarum* was successfully isolated from the cecum of piglets in a pig farm in Rongchang, Chongqing, China, and named LP-1 (GenBank accession no. MH727586.1). Its main metabolites were found to include acids (33.96%), amino acids (and their derivatives) (32.08%), and polysaccharides (15.1%) through the pristine analysis using gas chromatography–mass spectrometry (GC–MS) (Huang et al., 2021) LP, as a probiotic, also has the role of promoting intestinal health. Consequently, the present study aimed to investigate the inhibitory effect of *Lactiplantibacillus plantarum* supernatant (LP-1S) on PEDV and its mechanism.

## 2. Materials and methods

### 2.1. Cells, viruses, bacteria, reagents, and antibodies

African green monkey kidney cells (Vero, used at 10th generation) and porcine small intestinal epithelial cells (IPEC-J2, used at 10th generations) were purchased from Suer Biotech (Shanghai, China) and were cultured in Dulbecco’s modified Eagle’s medium (DMEM) medium with 10% fetal bovine serum, at 37°C, in a 5% CO_2_ cell incubator. PEDV-LJX strain and PEDV N mouse-derived monoclonal antibodies were kindly donated by GuangLiang Liu, Researcher, Lanzhou Veterinary Research Institute, Chinese Academy of Agricultural Sciences. *Lactiplantibacillus plantarum* LP-1 was obtained by pre-laboratory isolation. TRPV6 polyclonal rabbit antibodies (Vol: 50 μL, Cat No: 13411-1AP, Dilution: 1:4,000), horseradish peroxidase (HRP)-goat anti-mouse IgG (H + L) (Vol: 100 μL, Cat No: D110087-0100, Dilution: 1:4,000) and β-actin goat anti-rabbit antibody (Vol: 500 μL, Cat No: SA00001-2, Dilution: 1:5,000) were purchased from Proteintech. PMCA1b Polyclonal Rabbit Antibodies (Vol: 100 μL, Cat No: GR3312280-1, Dilution: 1:2,000) were purchased from Abcam (Cambridge, MA, United States). Fura-2-acetoxymethyl ester (Fura-2 AM) and CaCl_2_ were purchased from Beyotime Biotechnology Co. (Shanghai, China). EGTA, Fluo3-AM, and BAPTA-AM were purchased from Solarbio (Beijing, China). Bepridil hydrochloride (BP) was purchased from MCE (Monmouth junction, NJ, United States). Ca^2+^ standard solution (1,000 μg/mL) was purchased from National Research Center for Reference Materials, Beijing, China.

### 2.2. Preparation of LP-1S

*Lactiplantibacillus plantarum* LP-1 was inoculated onto MRS solid medium and incubated at 37°C. After 24 h, single colonies were picked, inoculated in MRS liquid medium, incubated at 37°C with shaking 180 rpm for 12 h until they reached a measured OD_595_ value between 0.8 ~ 0.9, and centrifuged at 3,000 × *g* for 10 min. The supernatant was retained and filtered using a 0.44 μm–0.22 μm microporous filter membrane gradient. The filtered supernatant (LP-S1) was stored at −20°C for a short time.

### 2.3. 3-(4,5-dimethylthiazol-2-yl)-2,5-diphenyltetrazolium bromide (MTT) assay

Firstly, LP-1S was diluted with DMEM basal medium to 0, 1/2, 1/4, 1/8, 1/16, 1/32, and 1/64 times the original concentration. Different dilutions of LP-1S were added to each well of a microtiter plate, containing 3.6 × 10^6^ cells per well. A positive control group (PEDV-infected group) and a negative control were set, and the adsorption culture was replaced with DMEM maintenance solution after 90 min. When the cytopathic effect (CPE) of the PEDV-infected group reached 70%, the maintenance solution was discarded, the wells were washed three times with 0.01 M phosphate-buffered saline (PBS), 5 mg/mL MTT reagent was added to each well, the plate was incubated at 37°C for 4 h to allow the formation of formazan crystals, and the OD_575_ values were then measured. Cell viability was calculated, and the IC50 of LP-S1 was calculated: lgIC50 = Xm-I (P- (3-Pm-Pn)/4) [in which Xm: lg maximum dose, I: lg (maximum dose/adjacent dose), P: sum of positive response rates, Pm: maximum positive response rate, Pn: minimum positive response rate].

### 2.4. TCID_50_ PEDV virulence assay

Cell samples from LP-1S pretreated PEDV-infected, PEDV-infected, and negative control cell groups were collected at different time points and centrifuged to collect supernatants. Vero cells in 96-well plates were grown to 90%, and different dilution gradients of LP-1S were inoculated into 96-well plates containing DMEM basal medium, and after the viruses were incubated separately for 1.5 h, the DMEM basal medium was replaced and the CPE was observed daily. Finally, the TCID_50_ of each PEDV group was measured according to the Reed-Muench method (Zhang et al., 2012).

### 2.5. Western blotting assay

Quantified protein samples were subjected to sodium dodecyl sulfate-polyacrylamide gel electrophoresis, then the separated proteins were transferred to polyvinylidene fluoride membranes (Merck Millipore, Billerica, MA, United States), which were blocked in 5% skim milk for 1.5 h, followed by incubation with the corresponding primary antibodies overnight at 4°C. Next day, the membranes were incubated with the corresponding secondary antibodies for 90 min at 37°C. The immunoreactive protein bands were visualized using the FX5 imaging system (VILBER, Marne-la-vallée, Ile-de-France, France), and the grayscale values were analyzed.

### 2.6. Quantitative real-time reverse transcription PCR

#### 2.6.1. RNA extraction and reverse transcription

Total RNA was extracted from cells and reverse transcribed to cDNA using RNAiso plus (Invitrogen, Waltham, MA, United States) and 5 × PrimeScript RT Master Mix (Promega, Madison, WI, United States).

#### 2.6.2. Relative fluorescence quantitative PCR

The full sequences of *TRPV6*, *PMCA1b*, and *ACTB* (β-actin genes) were downloaded from GenBank. Primers were designed against the sequences using Primer Premier 5.0 software (Premier Biosoft, San Francisco, CA, United States). The primer sequences are shown in Table 1. The cDNA was amplified using quantitative real-time PCR (qPCR) using ACTB as an internal reference by Biotech Co. Ltd., Shanghai, China. The reaction system comprised: 10 μL SYBR PreMix ExTaq II (Takara, Shiga, Japan), 0.5 μL forward primer, 0.5 μL reverse primer, 2 μL cDNA, and 7 μL H2O, in a 20 μL reaction. The reaction conditions were: 95°C for 30 s; followed by 40 cycles of 95°C for 5 s and 60°C for 15 s. Each sample was repeated three times. The primers are shown in in Table 1. The 2^−ΔΔCt^ method was used to analyze the expression of the corresponding mRNAs (Livak and Schmittgen, 2001).

**Table 1:** Primer sequences for *TRPV6*, *PMCA1b*, and *ACTB* (β-actin).

Gene	GenBank accession	Sequences (5′–3′)
*TRPV6*	CP071569.1	F: CCTGCTGGAACTTCTTGTCACCTC
		R: AAGTACCGCCGCCCGTATCTC
*PMCA1b*	X53456.1	F: CCTGCTGGAACTTCTTGTCACCTC
		R: CTGCTCCTGCTCAATTCGACTCTG
*ACTB*	XM_003124280.5	F: CTCTTCCAGCCCTCCTTCC
		R: GGTCCTTGCGGATGTCG

#### 2.6.3. Absolute fluorescence quantitative PCR

The PEDV-M primer sequences were downloaded from GenBank (Table 2). The PEDV gene copy number was detected using absolute fluorescence quantitative PCR. The reaction parameters were: pre-denaturation at 95°C for 30 s; followed by 40 cycles of denaturation at 95°C for 5 s, annealing at 54°C for 30 s, and 40 cycles. The linear relationship between cycle threshold (CT) values and copy number was analyzed using the Bio-Rad CFX Manager software random matrix method to calculate the copy number of the *PEDV M* gene.

**Table 2:** Primer sequences for the *PEDV M* gene.

Gene	GenBank accession	Sequences (5′–3′)
*PEDV M*	AF353511.1	F: AGGTTGCTACTGGCGTACAG
		R: GAGTAGTCGCCGTGTTTGGA

#### 2.6.4. Transfection with TRPV6 and PMCA1b interference plasmids

**Figure 1:**
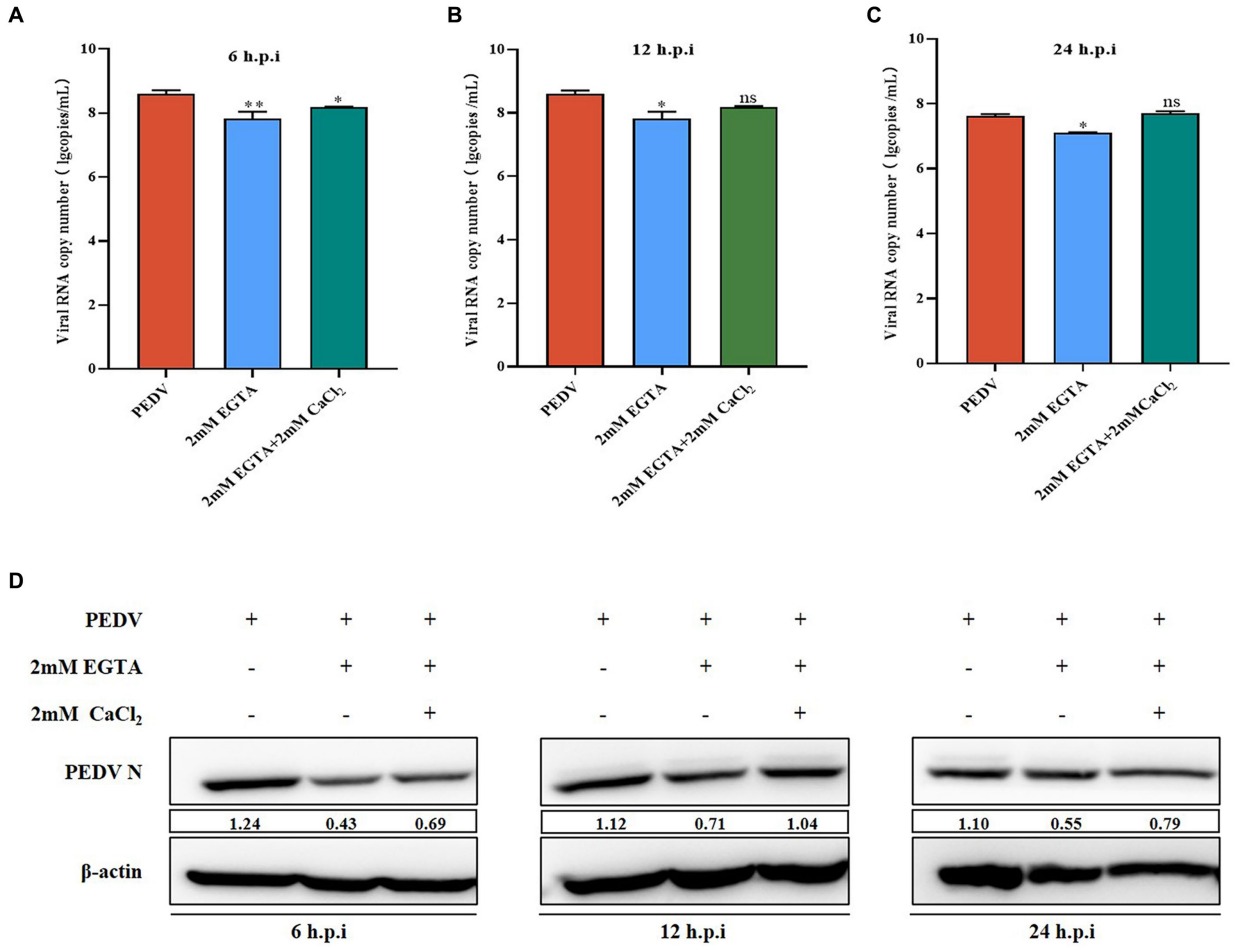
Effect of EGTA and CaCl_2_ pretreatment on PEDV. **(A)** PEDV-M mRNA levels at 6 h after EGTA and CaCl_2_ pretreatment in PEDV-infected cells; **(B)** PEDV-M mRNA levels at 12 h after EGTA and CaCl_2_ pretreatment in PEDV-infected cells; **(C)** PEDV-M mRNA levels at 24 h after EGTA and CaCl_2_ pretreatment in PEDV-infected cells; **(D)** PEDV-N protein levels at different time points after EGTA and CaCl_2_ pretreatment in PEDV-infected cells.

The shRNA sequence was designed and synthesized by Wuhan Jinkairui Biotechnology Co. (Figure 1) and then ligated with the interference vector pLVX-shRNA2Puro. 3.6 × 106 cells were inoculated in each well, and the cells were removed from the incubator when they reached 90% confluence at the time of transfection. Add 0.8 μg of plasmid DNA into 50 μL Opti-MEM medium and mix gently. Add 1.6 μL of TransIntroM EL into the diluted plasmid DNA, mix gently, and let it stand at room temperature for 15 min. Add the plasmid DNA-TransIntromM EL complex into the cells and incubate at 37°C CO2 for 6 h. After transfection, remove the cells from the incubator. The cells were incubated at 37°C in a CO2 incubator, and after 6 h of transfection, the medium was changed and the culture was continued for 36 h.

### 2.7. Calcium ion fluorescent probe (Fluo3-Am) analysis

Fluo3-AM was diluted to a 2 mM Fluo3-AM stock solution using dimethyl sulfoxide and stored at −20°C for later use. The preserved Fluo3-AM stock solution was diluted with 0.1 M Hanks’ Balanced Salt Solution (HBSS; Beyotime Biotechnology Co.), added to cells in a microtiter plate (3.6 × 10^6^ cells per well) and incubated for 60 min at 37°C with 5% CO_2_. The wells were washed using 0.1 M HEPES (Amphoteric buffer, Beyotime Biotechnology Co.) buffered saline three times, added with HBSS containing 1% fetal bovine serum, and incubated for 30 min. Changes in intracellular calcium ion content were observed using fluorescence microscopy.

### 2.8. Flame atomic absorption analysis

0.25 mL, 0.5 mL, 1 mL, 1.5 mL, and 2 mL of a Ca standard solution (10 μg/mL) were added to a 50 mL volumetric flask and added with 50 mL with medium solution (0.5% HNO_3_ + 2 mg/mL K^+^). Then, the standard concentration was measured and the standard curve was plotted. Finally, the collected cell supernatant and intracellular fluid were diluted 5,000 times with medium solution and then assayed on the flame atomic absorption spectrophotometer. The absorbance and concentration values of each sample were recorded. Changes in extracellular calcium ion content were then analyzed with reference to the standard curve.

### 2.9. Statistical analysis

All results were subjected toa *t*-test and one-way analysis of variance (ANOVA) using the statistical software GraphPad Prism 8.0 (GraphPad Inc., La Jolla, CA, United States) and IBM SPSS Statistics 23 to determine statistical differences between multiple groups. A *p*-value > 0.05 (ns) indicates a non-significant difference, a *p*-value < 0.05 indicates a significant difference (*), a *p*-value < 0.01 expresses a highly significant difference (**), and a *p*-value < 0.001 expresses a very significant difference (***).

## 3. Results

### 3.1. PEDV infection affects changes in intra- and extracellular Ca^2+^ concentrations

**Figure 2:**
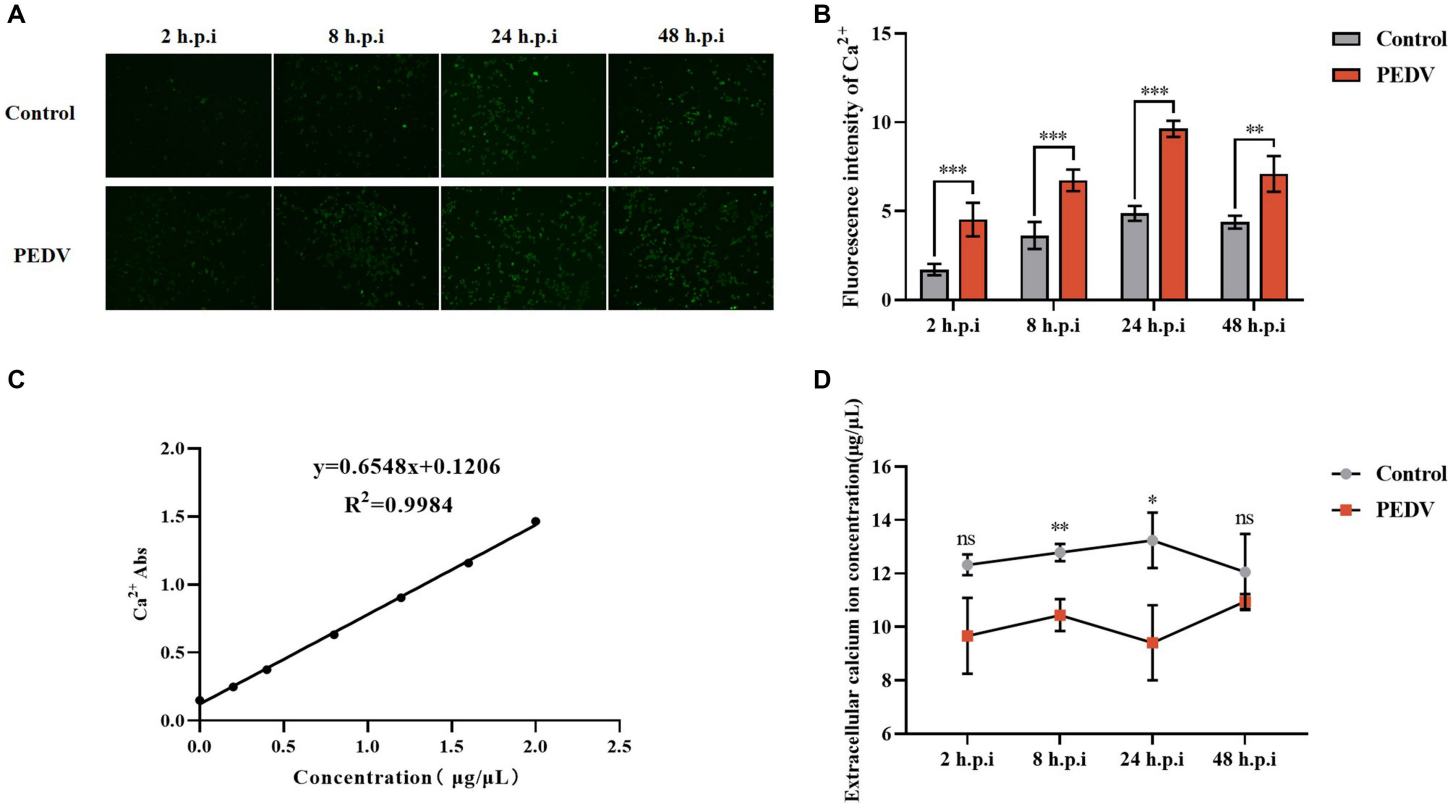
Changes in intra- and extracellular Ca^2+^ concentrations after PEDV infection. **(A)** intracellular Ca^2+^ fluorescence intensity graph (10×); **(B)** fluorescence intensity statistics graph; **(C)** Ca^2+^ standard curve; **(D)** extracellular Ca^2+^ concentration measurement.

Several viruses have been shown to promote their replication and infection by modulating cellular calcium ion levels; however, the effect of PEDV infection on intra- and extracellular Ca^2+^ concentrations is unknown. We used the calcium ion fluorescence probe Fluo-3 AM to detect the intracellular Ca^2+^ concentration under PEDV infection, which showed that the intracellular Ca^2+^ fluorescence intensity after 2 h, 8 h, 24 h, and 48 h of PEDV infection was extremely significantly higher than that in the negative control group, under fluorescence microscopy (Figures 2A,B). The changes in the extracellular Ca^2+^ concentration under PEDV infection were detected using the flame atomic absorption method. A Ca^2+^ standard curve was first established (Figure 2C). The results of sample detection showed that the Ca^2+^ concentration in the extracellular fluid was significantly lower after PEDV infection (at 2 h, 8 h, 24 h, and 48 h) compared with that in the negative control group, in which the calcium ion concentration in the extracellular fluid was significantly lower in the 2 h, 8 h, and 24 h infection groups compared with that in the negative control group (Figure 2D). These results showed that PEDV infection causes a significant difference in the intra- and extracellular Ca^2+^ concentrations, i.e., PEDV infection causes a significant increase in the flow of Ca^2+^ from the extracellular space into the cells.

### 3.2. Inhibition of PEDV replication by the divalent cation chelating agent EGTA

**Figure 3:**
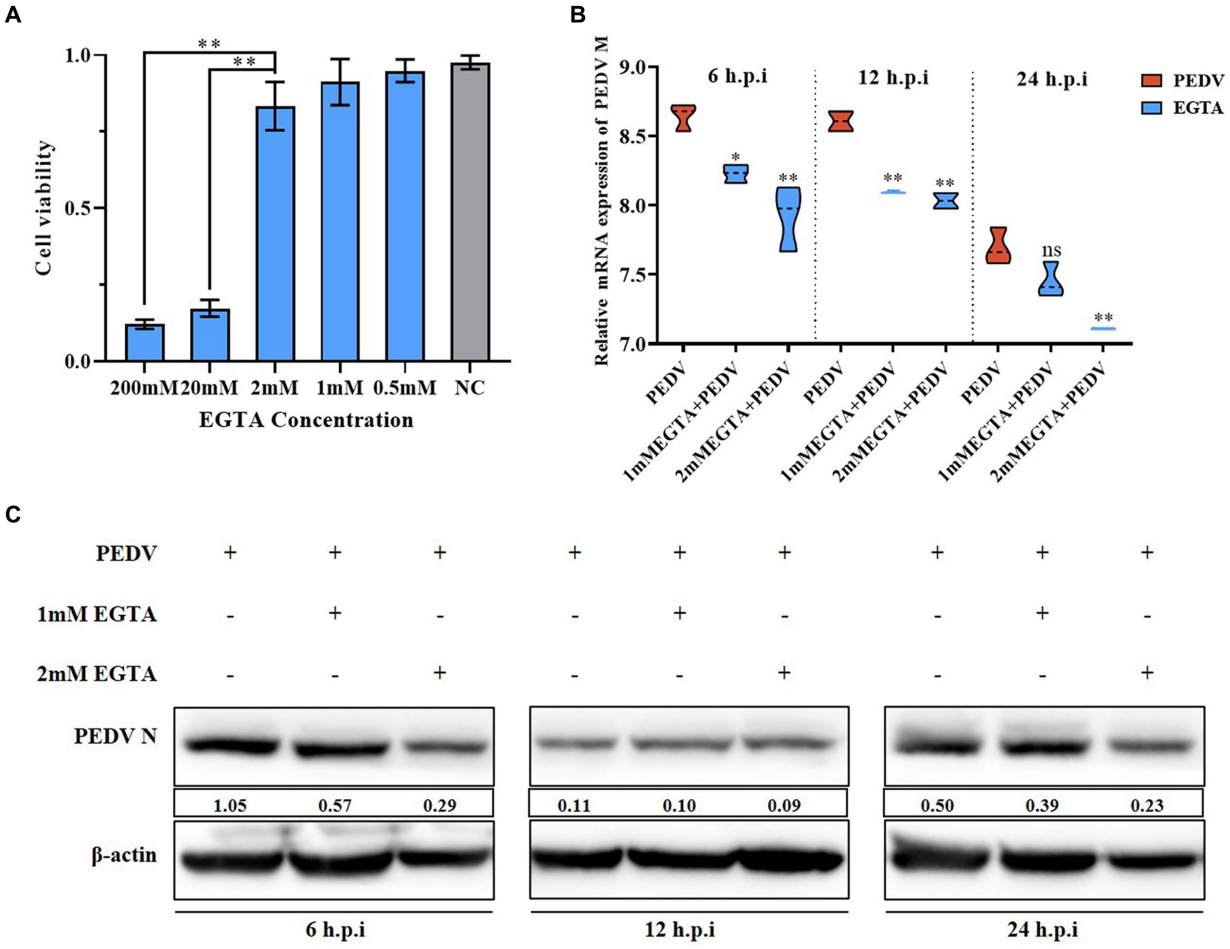
Effect of different concentrations of EGTA on PEDV. **(A)** EGTA toxicity assay in IPEC-J2 cells; **(B)** PEDV M gene copy number at 6 h, 12 h, and 24 h after pretreatment of cells with different concentrations of EGTA followed by PEDV infection; **(C)** PEDV-N protein levels at 6 h, 12 h, and 24 h after pretreatment of cells with different concentrations of EGTA followed by PEDV infection.

We observed that PEDV infection can cause significant changes in intra- and extracellular Ca^2+^ concentrations. To further elucidate the relationship between Ca^2+^ and PEDV, we treated the cells with the specific Ca^2+^ chelator, EGTA, to detect the effect of Ca^2+^ on PEDV replication. First, the toxicity of EGTA toward IPEC-J2 cells was examined using the MTT assay, which showed that an EGTA concentration below 2 mM was not toxic to IPEC-J2 cells (Figure 3A). qRT-PCR and western blotting were used to detect the effect on PEDV replication after pretreatment of IPEC-J2 cells with different concentrations of EGTA. The results showed that after pretreatment with 1 mM and 2 mM EGTA, the PEDV M gene copy number decreased significantly at 6 h, 12 h, and 24 h post-infection compared with that in the untreated group, and the effect of 2 mM EGTA was more obvious (Figure 3B). The results of western blotting also showed that the PEDV-N protein level decreased at 6 h, 12 h, and 24 h after 1 mM and 2 mM EGTA pretreatment compared with the PEDV-infected only group, and the effect of 2 mM EGTA pretreatment at 6 h and 24 h was more obvious (Figure 3C). This demonstrated that EGTA pretreatment had an inhibitory effect on PEDV in a concentration-dependent manner. However, since EGTA can also chelate other divalent cations in the cell, whether the inhibition of PEDV replication in this process is dependent on Ca^2+^ chelation needs to be further investigated.

### 3.3. Supplementation of extracellular Ca^2+^ concentration to promote PEDV infection

In Section 3.2, we demonstrated that EGTA pretreatment inhibited PEDV replication. To confirm that EGTA exerts its effect by chelating Ca^2+^, we first added CaCl_2_ solution after EGTA pretreatment and examined the changes of PEDV after Ca^2+^ concentration backfill. The results showed that the PEDV-M copy number was significantly lower than that of the PEDV-infected only group after 6, 12, and 24 h after 2 mM EGTA pretreatment, while the PEDV-M copy number increased significantly after pretreatment in the 2 mM EGTA+CaCl_2_ group compared with that in the 2 mM EGTA pretreatment only group (Figure 1A). The PEDV-N protein level was also detected and found to be significantly lower in the 2 mM EGTA pretreatment group than in the PEDV-infected only group; while the PEDV-N level in the 2 mM EGTA+CaCl_2_ group was significantly higher than that in the EGTA group (Figure 1B).

### 3.4. Ca^2+^ channel inhibitors promote PEDV infection

**Figure 4:**
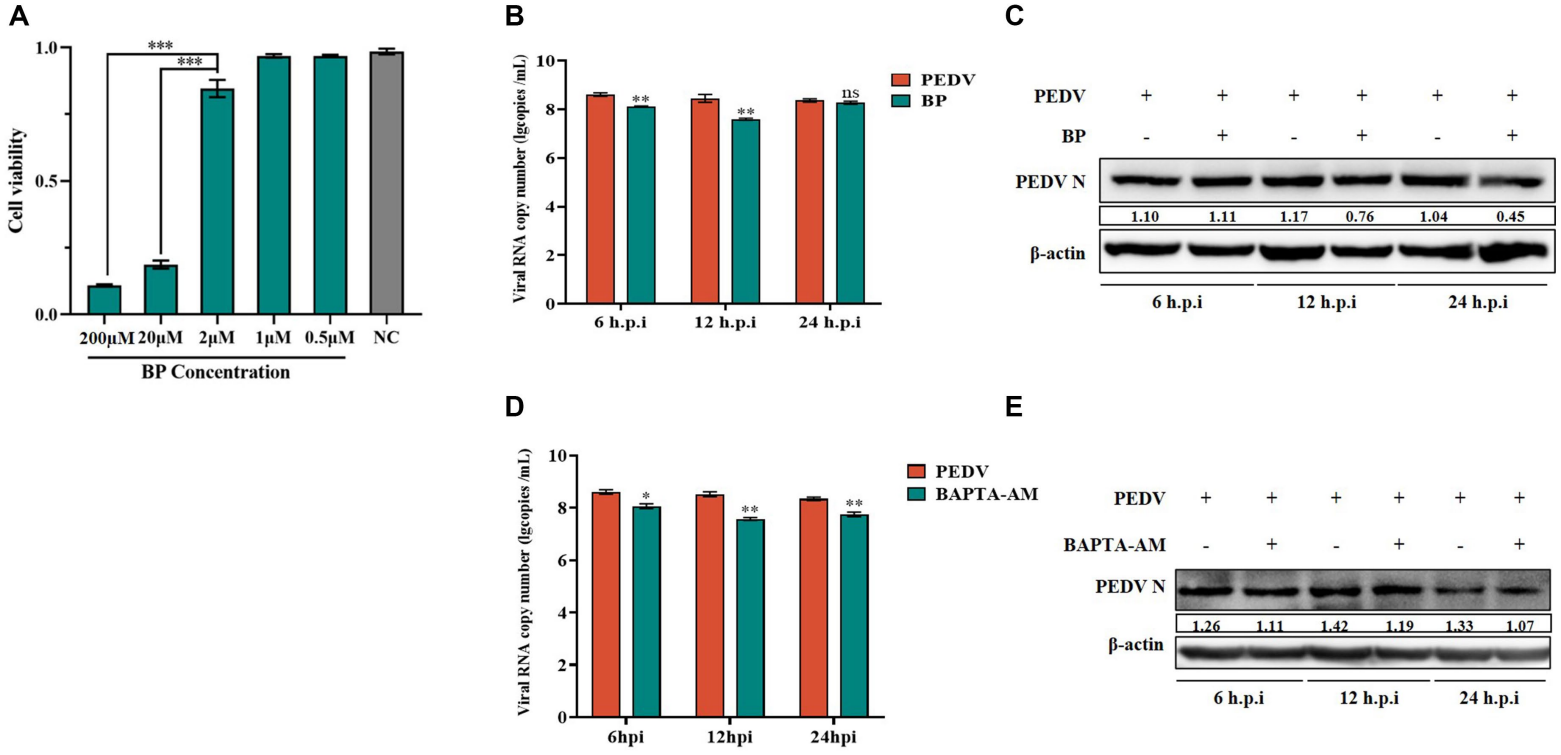
Effect of BP and BAPTA-AM on PEDV. **(A)** Cytotoxicity assay of BP; **(B)** PEDV M gene copy number in BP pretreated cells infected with PEDV at 6 h, 12 h, and 24 h; **(C)** PEDV N protein levels in the PEDV group after BP treatment; **(D)** PEDV M gene copy number in BAPTA-AM pretreated cells infected with PEDV at 6 h, 12 h, and 24 h; **(E)** PEDV N protein levels in the PEDV group after BAPTA-AM treatment.

To further investigate the specific role played by calcium channels during viral infection, we chose a long-acting calcium channel inhibitor (BP) and the Ca^2+^ complexing agent BAPTA-AM to investigate the role of calcium channels in PEDV infection. BP inhibits cellular Ca^2+^ inward flow. BAPTA is released from BAPTA-AM by the action of a lipase upon entry into the cell. The released BAPTA can rapidly complex with Ca^2+^, thus controlling the intracellular calcium ion levels. We detected no cytotoxicity of BP on IPEC-J2 cells at less than 2 μM using the MTT assay (Figure 4A). IPEC-J2 cells pretreated with 2 μM BP and then infected with PEDV infected were examined for PEDV-M gene copy number as well as PEDV N protein expression levels. The results showed that after 6–24 h of infection, the PEDV-M copy number was lower than that of the PEDV-infected only group, which was more significant at 6 h and 12 h (Figure 4B); the PEDV-N protein level was also reduced in the BP pretreated group, and the reduction was more significant after 12 h (Figure 4C). Thus, BP significantly inhibited PEDV M gene expression from 6 to 24 h, and the inhibitory effect on the PEDV-N protein level was more pronounced with extended time. In addition, we similarly observed a significantly lower PEDV-M gene copy number than that in the PEDV-infected alone group from 6 to 24 h after pretreatment with 20 μM BAPTA-AM (Figure 4D); the PEDV-N protein levels also decreased more significantly after 12 h (Figure 4E).

### 3.5. Important role of channel proteins in the regulation of intra- and extracellular Ca^2+^

**Figure 5:**
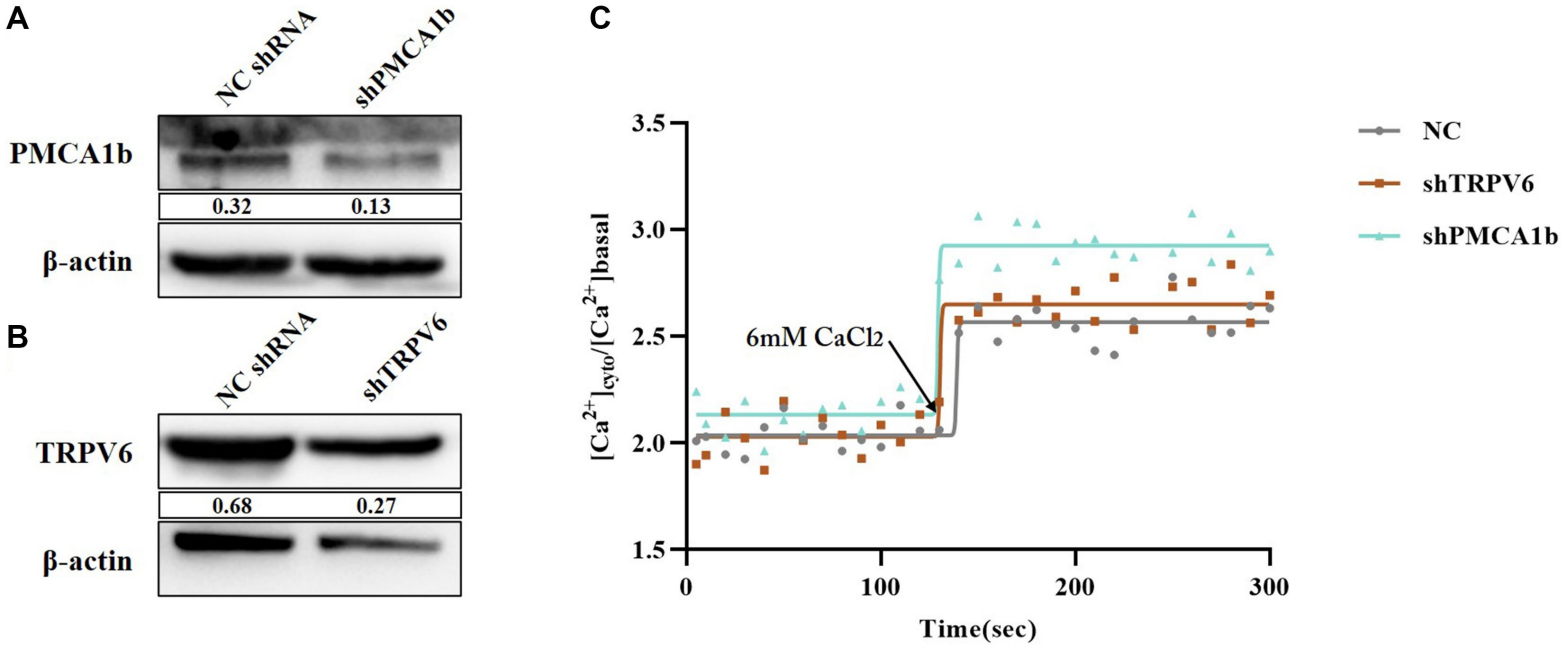
Validation of the effect of *TRPV6*/*PMCA1b* interference and its impact on intracellular Ca^2+^. **(A)** PMCA1b protein levels after shPMCA1b expression in IPEC-J2 cells; **(B)** TRPV6 protein levels after shTRPV6 expression in IPEC-J2 cells; **(C)** Effect of shTRPV6/shPMCA1b treatment on intracellular Ca^2+^.

Intracellular and extracellular Ca^2+^ transmembrane transport mainly involves membrane transporter proteins, carrier proteins, and channel proteins. To further investigate the role played by calcium channel proteins during PEDV infection, we selected two typical Ca^2+^ transport channels, TRPV6 and PMCA1b, as the subjects of our study. Firstly, we constructed vectors for RNA interference of *TRPV6* and *PMCA1b* (shTRPV6/shPMCA1b) separately to determine the association between TRPV6/PMCA1b and PEDV. The results showed that both interfering vectors were stably expressed in IPEC-J2 cells and significantly reduced the protein levels of TRPV6 and PMCA1b (Figures 5A,B). The fluorescence from the interference vector would have an effect on the detection of Fluo-3 AM; therefore, another calcium probe, Fura-2 AM, was chosen to detect the intracellular Ca^2+^ concentration. As shown in Figure 5C, the intracellular Ca^2+^ concentration was elevated after interfering with *TRPV6* and *PMCA1b* compared with that in the blank group, and the addition of 6 mM CaCl_2_ at 120 s showed a more pronounced upregulation of the Ca^2+^ concentration in the *PMCA1b* interference group (Table 3).

**Table 3:** Sequence of *TRPV6* and *PMCA1b* shRNA interference fragments.

Gene	Sequences (5′–3′)
*TRPV6*	F: 5′-GGTGGAAGACAGACAGGATAT-3′
*PMCA1b*	F: 5′-GGGCGCCTGTATTACTCAAGA-3′

### 3.6. Ca^2+^ channel proteins inhibit PEDV infection

**Figure 6:**
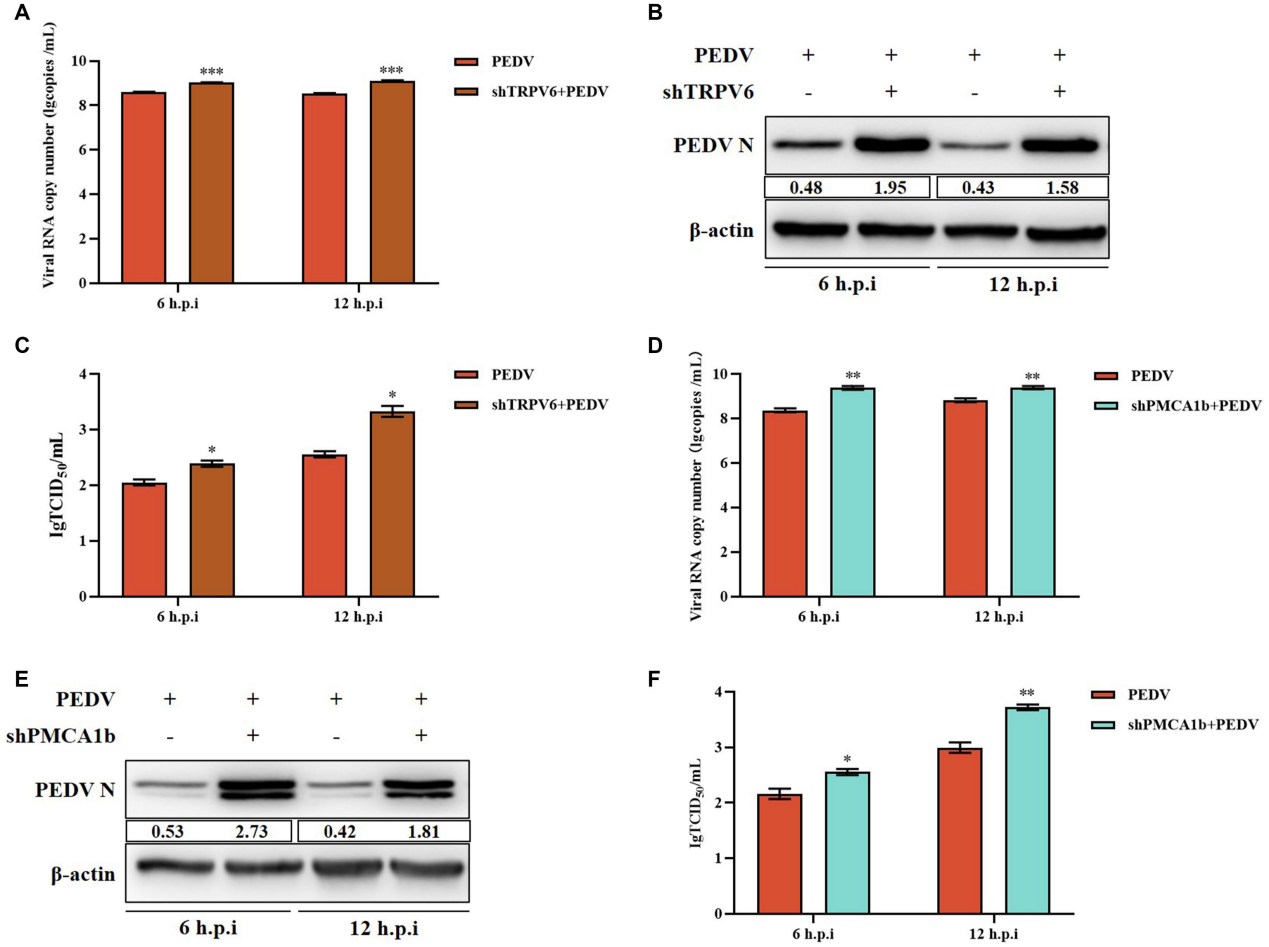
Effect of interfering with *TRPV6* and *PMCA1b* on PEDV. **(A)** PEDV-M gene copy number in IPEC-J2 cells after shTRPV6 treatment; **(B)** PEDV-N protein levels in IPEC-J2 cells after shTRPV6 treatment; **(C)** PEDV infection of IPEC-J2 after shTRPV6 pretreatment cells; **(D)** PEDV-M gene copy number in IPEC-J2 cells after shPMCA1b treatment; **(E)** PEDV protein levels in IPEC-J2 cells after shPMCA1b treatment; **(F)** change in the viral titer in IPEC-J2 cells infected with PEDV after shPMCA1b pretreatment.

Under normal physiological conditions, TRPV6 mediates Ca^2+^ inward flow and PMCA1b promotes intracellular Ca^2+^ flow to the extracellular space. After 6 h and 12 h post-PEDV infection, the PEDV-M copy number and PEDV-N protein levels were significantly upregulated in the shTRPV6 group, and the viral titer was also significantly increased (Figures 6A–C). Similarly, the PEDV-M gene copy number, PEDV-N protein level, and the viral titer were significantly increased in the shPMCA1b group (Figures 6D–F).

### 3.7. LP-1S can effectively inhibit PEDV replication

**Figure 7:**
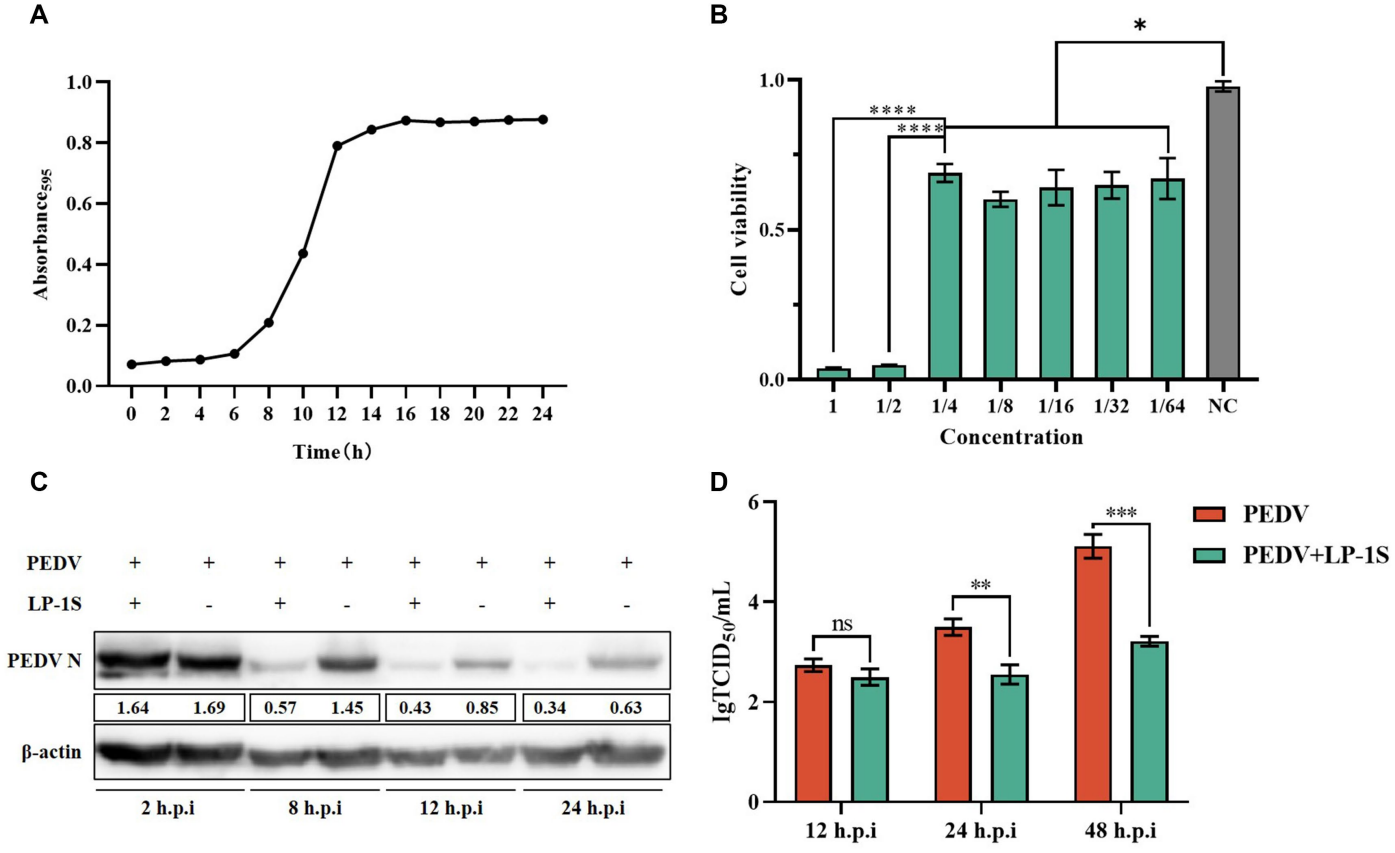
LP-1S can effectively inhibit PEDV replication **(A)** Growth curve of *Lactiplantibacillus plantarum* under LP-1S treatment. **(B)** MTT assay detection of the toxicity of LP-1S toward IPEC-J2 cells; **(C)** PEDV-N levels after treatment with 1/4 times dilution of LP-1S; **(D)** TCID50 results after LP-1S pretreatment.

*Lactobacillus*, as a probiotic flora, is highly effective against intestinal diarrheal diseases. Probiotics are present in the intestinal tract of animals and have a protective effect on intestinal health. Our laboratory previously isolated a strain of *Lactiplantibacillus plantarum*, LP-1, and we sought to verify whether the metabolites of this bacterium have an anti-PEDV effect. We first measured its growth curve, and the results showed that LP-1 entered the logarithmic growth phase after 6 h and reached the end of logarithmic growth phase (maximum growth value) at 12 h (Figure 7A); therefore, we chose 12 h as the best time to obtain the *Lactiplantibacillus plantarum* supernatant, LP-S1. Subsequently, the toxicity of LP-S1 toward IPEC-J2 cells was determined using the MTT assay, and the LP-1S 1/4-fold dilution was found to be the optimal treatment concentration dose for IPEC-J2 cells (Figure 7B). The 1/4-fold dilution of LP-1S was used to pretreat the cells in the PEDV-infected group, and the proteins were collected at 2 h, 8 h, 12 h, and 24 h. Western blotting showed that the PEDV-N protein level in the LP-1S pretreatment group decreased gradually and significantly with increasing time (Figure 7C). The viral solution collected at different time points after LP-1S pretreatment was used to detect the viral titer, and the TCID50 data showed that the viral titer at 12 h, 24 h, and 48 h showed a decreasing trend, with a more significant decrease at 24 h (Figure 7D).

### 3.8. LP-1S upregulates the Ca^2+^ channel protein

**Figure 8:**
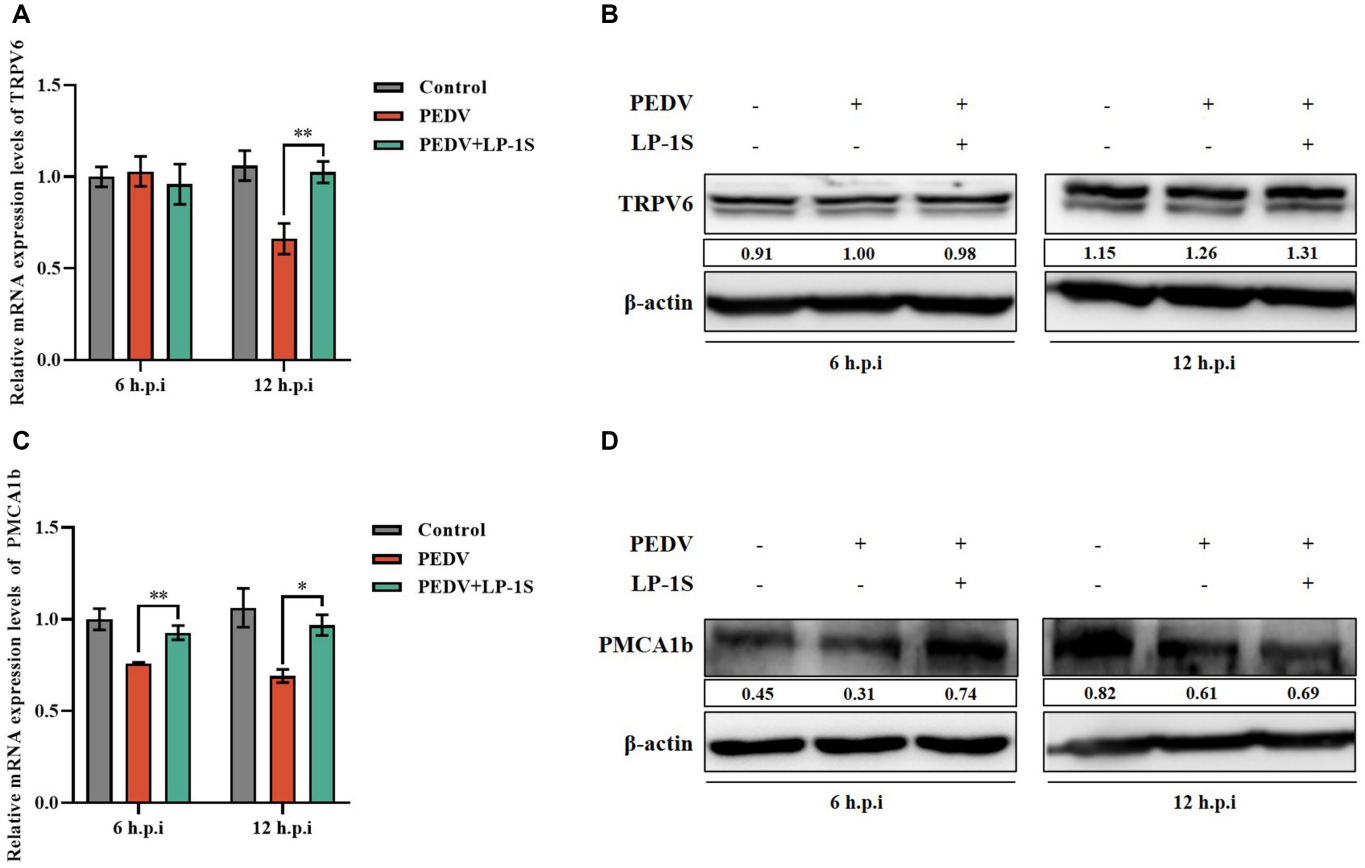
The level of expression of TRPV6 and PMCA1b after LP-1S treatment. **(A)** *TRPV6* mRNA statistics after attack following LP-1S treatment; **(B)** TRPV6 protein expression levels after attack following by LP-1S treatment; **(C)** *PMCA1b* mRNA statistics after attack followingLP-1S treatment; **(D)** PMCA1b protein levels after attack following LP-1S treatment.

We showed that PEDV infection caused elevated intracellular Ca^2+^ and was associated with two Ca^2+^ channel proteins, TRPV6 and PMCA1b. LP-1S has an anti-PEDV effect; however, whether it affects Ca^2+^ is unknown. TRPV6 and PMCA1b are calcium channel proteins mainly expressed in the gastrointestinal tract and have high permeability to Ca^2+^. To further verify whether LP-1S is associated with Ca^2+^ channel proteins, we examined the mRNA and protein expression levels of TRPV6 and PMCA1b in the PEDV infection group and after LP-1S pretreatment. The results showed that the mRNA level of *TRPV6* significantly increased at 12 h after LP-1S pretreatment compared with that in the PEDV-infected only group (Figure 8A), and the protein level likewise significantly increased at 12 h (Figure 8B). Similarly, the mRNA and protein levels of PMCA1b increased after LP-1S pretreatment compared with those in the PEDV-infected group, with mRNA levels at 6 h and 12 h (Figure 8C) and protein levels at 6 h (Figure 8D) being the most significant.

### 3.9. LP-1S affects changes in intra- and extracellular Ca^2+^ concentrations

**Figure 9:**
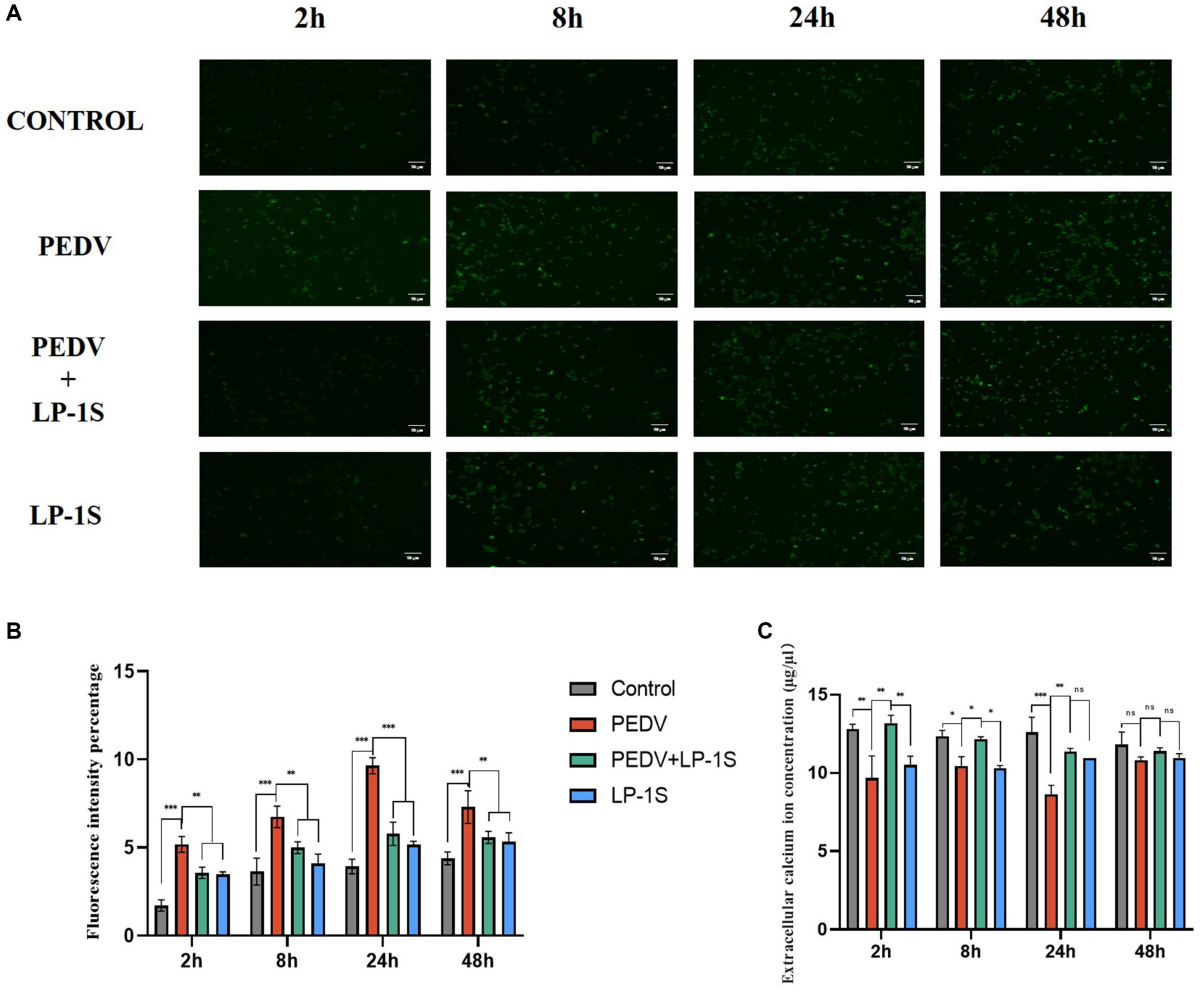
Effect of tapping on intra- and extracellular calcium ions after LP-1S treatment. **(A)** Fluorescence plot of intracellular calcium ions; **(B)** fluorescence statistics of intracellular calcium ions; **(C)** Changes in extracellular Ca^2+^.

It was previously verified that Ca^2+^ endocytosis contributes to PEDV infection, and TRPV6 and PMCA1b are key channel proteins that regulate Ca^2+^. LP-1S pretreatment significantly reduced the extent of PEDV infection and increased the levels of TRPV6 and PMCA1b. To further investigate the relationship between LP-1S and Ca^2+^, we examined the changes induced by LP-1S on intra- and extracellular Ca^2+^ concentrations using the calcium fluorescent probe Fluo-3 AM and flame atomic absorption, respectively. According to the intracellular Ca^2+^ concentration, the intensity of intracellular Ca^2+^ fluorescence was significantly weaker in the negative control group, the LP-1S pre-treatment group, and the LP-1S group than in the PEDV-infected group, and the intracellular Ca^2+^ concentration was significantly lower than that of the PEDV-infected group at 2 h, 8 h, 24 h, and 48 h (Figures 9A,B). In terms of the extracellular Ca^2+^ concentration, the extracellular Ca^2+^ concentration after LP-1S pretreatment was higher than that of the PEDV-infected group at 2 h, 8 h, 24 h, and 48 h, with 2–24 h being the most significant period (Figure 9C).

## 4. Discussion

In recent years, despite the increased research on PEDV in various countries, the incidence of PEDV-induced diarrhea in piglets remains high in clinical practice; therefore, the search for effective anti-PEDV drugs is still a top priority for PEDV prevention and control. Calcium ion imbalance is a hallmark of viral infection (Kumar et al., 2022). Several studies have shown that different viral infections lead to an imbalance of calcium ions inside and outside the cell. For example, the mRNA encoding the calbindin D-28 K and calretinin has an important role in the early stages of rabies infection (Korie et al., 2023). SARS-CoV-2 ORF 3a interferes with calcium homeostasis and induces autophagy to enhance viral infection (Garrido-Huarte et al., 2023). Calcium imbalance triggers the mitochondrial apoptotic pathway to promote infection by chicken anemia virus (Yu et al., 2023). Herpes simplex virus (HSV) infection induces a rapid increase in the intracellular calcium ion concentration, which plays a key role in facilitating virus entry (Jiang et al., 2023). Rotavirus disrupts calcium homeostasis by NSP4 viroporin activity, directly or indirectly, in response to elevated cytoplasmic calcium levels and regulates rotavirus replication and virion assembly (Hyser et al., 2010). Dengue virus (DENV) infection disrupts intracellular calcium homeostasis causing an increase in intracellular calcium levels (Dionicio et al., 2018).

We found that PEDV infection activates plasma membrane Ca^2+^ channels, leading to a large accumulation of intracellular calcium ions, and that this Ca^2+^ imbalance leads to changes in intracellular gene expression, energy metabolism, and other functions, which in turn promotes viral replication (Figure 6). We then further verified the link between PEDV and Ca^2+^ using a divalent cation chelating agent (EGTA), calcium channel inhibitors (BP and BAPTA-AM), and calcium channel proteins (TRPV6 and PMCA1b). Pretreatment with 2 mM EGTA significantly inhibited PEDV M expression, and PEDV expression M was significantly increased when the cells were supplemented with Ca^2+^, suggesting that EGTA effectively reduced the inward flow of extracellular free Ca^2+^ by chelating it, thereby inhibiting PEDV proliferation. In contrast, CaCl_2_ supplementation induced an increase in intracellular Ca^2+^ flow, leading to an increase in PEDV expression (Figure 1). Similarly, PEDV infection was significantly reduced when Ca^2+^ endocytosis was blocked using BAPTA-AM. In addition, the calcium channel protein TRPV6, which strictly regulates Ca^2+^ influx, is necessary to avoid Ca^2+^ overload (McGoldrick et al., 2018). When we interfered with *TRPV6* expression, PEDV invasion was significantly enhanced, which might have been caused by the disruption of Ca^2+^ channel function, resulting in intracellular Ca^2+^ overload, thus facilitating the viral invasion. By contrast, interfering with P*MCA1b* significantly inhibited its associated channels, reducing the flow of intracellular Ca^2+^ into the extracellular compartment, thereby exacerbating PEDV invasion. Therefore, we concluded that PEDV infection can be significantly reduced by inhibiting calcium channels, and this effect gradually increases with time (Figure 6).

**Figure 10:**
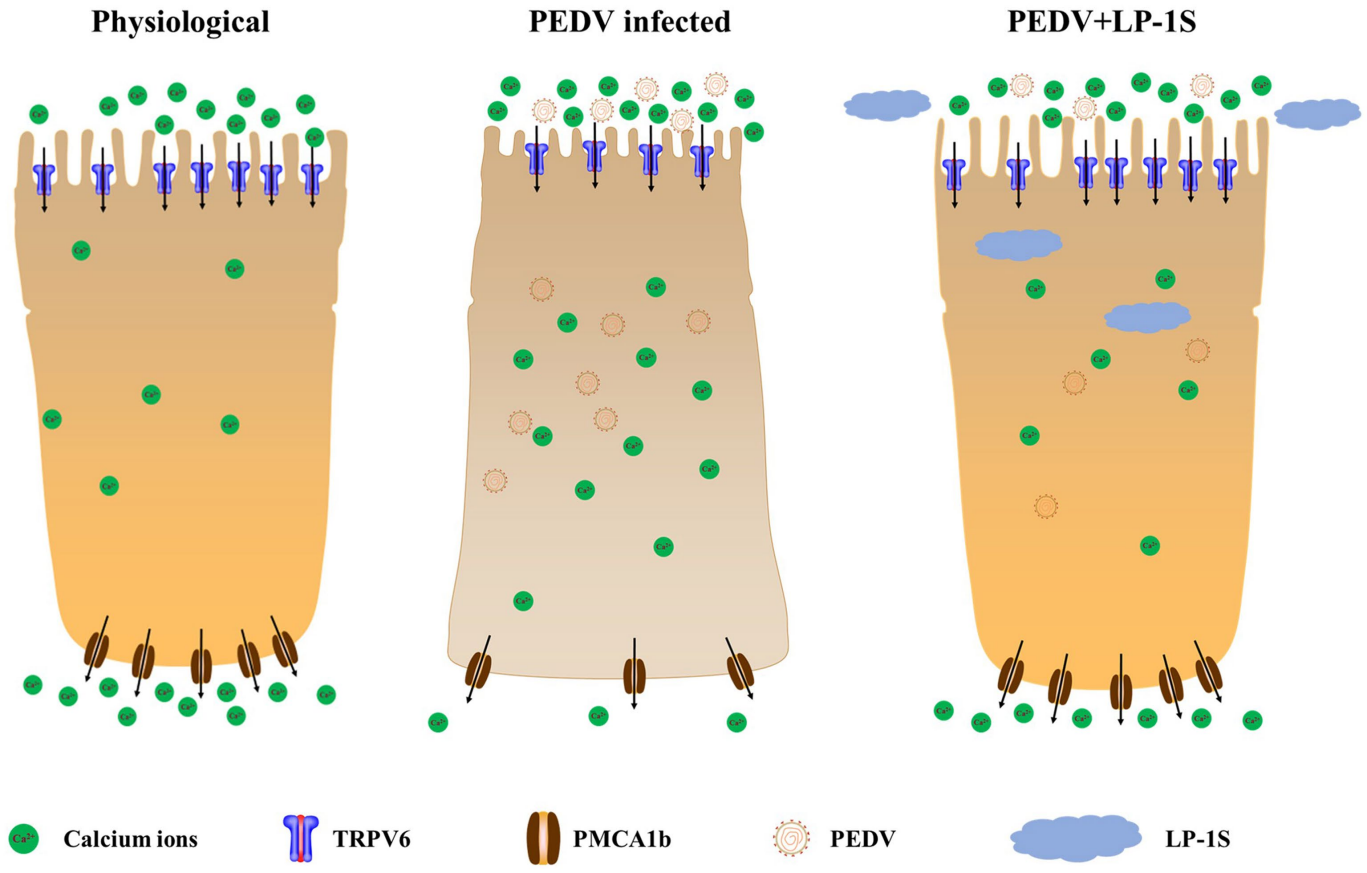
The mechanism by which LP-1S regulates Ca^2+^.

Probiotics are now considered as an effective alternative to antibiotics, and can colonize the intestinal tract. Probiotics function by reducing inflammatory response, protecting the intestinal structure, maintaining intestinal microbial homeostasis, and improving the immunity of the body (Thomas and Versalovic, 2010). The metabolites in LP-1S inhibited PEDV proliferation in a time- and concentration-dependent manner; however, whether this effect correlated with Ca^2+^ concentration has not been reported. To further reveal whether LP-1S has the ability to modulate Ca^2+^ concentration and thus exert antiviral effects, we conducted a follow-up study. The results showed that LP-1S could significantly reduce the intracellular Ca^2+^ concentration. A low intracellular Ca^2+^ concentration is a prerequisite to ensure normal cell function therefore, this might be an important method by which LP-1S inhibits the proliferation of PEDV. Further study revealed that under PEDV infection conditions, LP-1S pretreatment affected intestinal Ca^2+^ channel proteins (TRPV6, PMCA1b), among which PMCA1b was more sensitive to PEDV infection than TRPV6. LP-1S might precisely enhance the regulation of Ca^2+^ by PMCA1b and TRPV6 to reduce the intracellular Ca^2+^ concentration and maintain an intracellular low calcium state, thus exerting an anti-PEDV effect (Figure 10). Therefore, intracellular calcium homeostasis is particularly important to maintain normal cellular physiological functions and resistance to viral infection (Klapczyńska et al., 2023; Sørensen et al., 2023). Overall, LP-1S significantly reduced the intracellular Ca^2+^ concentration, whereas the increase in the extracellular Ca^2+^ concentration suggested that LP-1S might exert its anti-PEDV effects mainly through Ca^2+^ channel proteins that inhibit intracellular Ca^2+^ in-flow and promote Ca^2+^ out-flow (Figure 9).

In this study, we used calcium ion modulators to determine that an intra- and extracellular Ca^2+^ imbalance promotes PEDV infection. Using western blotting and qRT-PCR, LP-1S was observed to significantly inhibit PEDV replication. Finally, using Ca^2+^ fluorescent probes and flame atomic absorption, we demonstrated that LP-1S ameliorates the PEDV-induced Ca^2+^ imbalance. These result provides a possible treatment strategy for PEDV and could from the basis for the development of effective drugs.

## Data availability statement

The datasets presented in this study can be found in online repositories. The names of the repository/repositories and accession number(s) can be found in the article/supplementary material.

## Ethics statement

Ethical approval was not required for the studies on animals in accordance with the local legislation and institutional requirements because only commercially available established cell lines were used.

## Author contributions

ZK, SZ, and GL: conceptualization. ZK: methodology. ZN: software. ZK, SZ, and ZN: validation. ZSu and XL: formal analysis. XH: investigation. ZSo: resources, supervision, project administration, and funding acquisition. YZ: data curation. SZ: writing—original draft preparation. ZN: writing—review and editing. SX, JZ, and HZ: visualization. All authors contributed to the article and approved the submitted version.
